# Test your knowledge and understanding

**Published:** 2012

This page is designed to test your understanding of the concepts covered in this issue and to give you an opportunity to reflect on what you have learnt.

## Diagnose This: quiz 1

**Figure:**
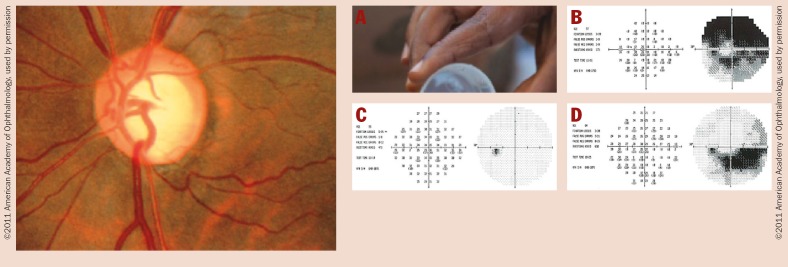


The photograph on the left shows the left optic nerve of one of your patients. Which of the four visual field tests (above, right) would best match this photograph: A, B, C, or D?

## Diagnose This: quiz 2

**Figure:**
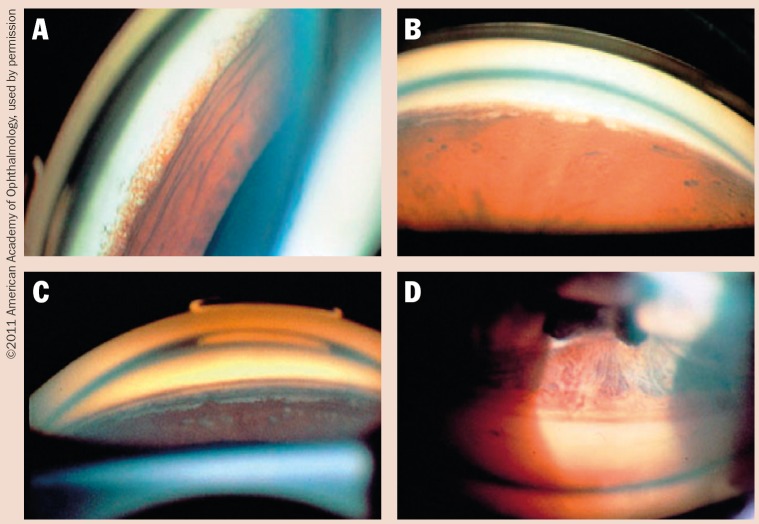


Which of the gonioscopic photographs above would represent a normal anatomic finding: A, B, C, or D?

## Diagnose This: quiz 3

**Figure:**
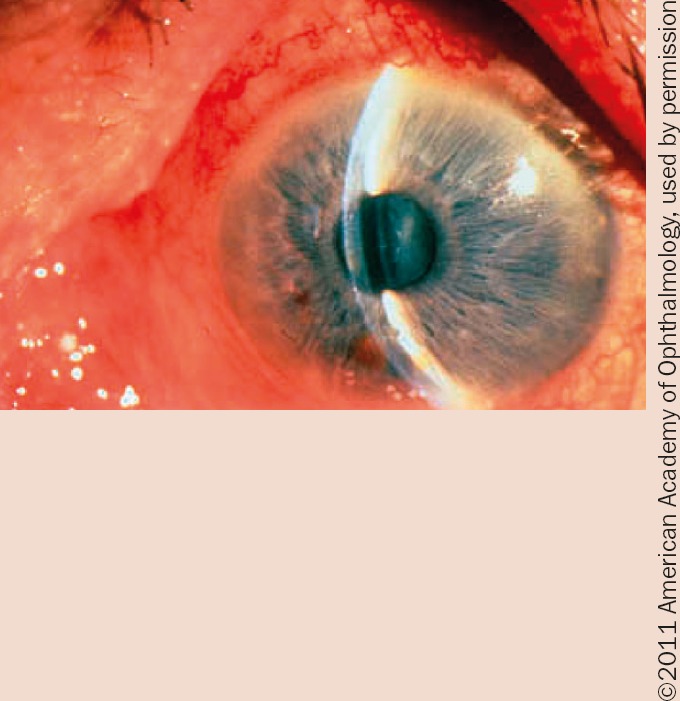


A patient with primary open-angle glaucoma underwent trabeculectomy. On the first post-operative day, the visual acuity corrected to 20/80, the bleb was almost flat, the anterior chamber shallow, and the intraocular pressure was 1 mmHg. What is the most likely problem?

- Aqueous misdirection (malignant or ciliary-block glaucoma)
- Ciliary body shutdown
- Early failure of bleb with scarring at episcleral surface
- Bleb leak

## ANSWERS

### Quiz 1

The left optic nerve depicted in the photograph has an inferotemporal thinning of the optic nerve rim and notch formation from the 5 o'clock to 6 o'clock position. **The superior visual field loss in Figure a would match this optic nerve damage**. The visual field in Figure B would require an optic nerve with advanced damage of the superior and inferior neural-retinal rim. The visual field in Figure C is normal. The visual field in Figure D would be found in a patient with a defect in the superior portion of the optic nerve.

### Quiz 2

**Figure A represents a normal finding** and shows a heavy layer of uveal trabecular meshwork, or iris processes. Figure B shows scattered peripheral anterior synechiae in an eye with previous episodes of acute anterior uveitis. Figure C shows traumatic angle recession, and Figure D shows rubeotic vessels in the angle on the trabecular meshwork of a patient with proliferative diabetic retinopathy.

### Quiz 3

**The most likely problem is bleb leak**. In the immediate post-operative period after trabeculectomy for primary open-angle glaucoma, the most common reason for a shallow anterior chamber with a low intraocular pressure and low bleb is a wound leak. Early bleb failure and aqueous misdirection could be suspected if the intraocular pressure was elevated after surgery. Ciliary body shutdown can occur but would be less likely than an unrecognised bleb leak in this clinical situation.

